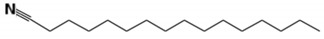# MEG Effects on Hydrolysis of Polyamide 66/Glass Fiber Composites and Mechanical Property Changes

**DOI:** 10.3390/molecules24040755

**Published:** 2019-02-20

**Authors:** Jong-Young Lee, Kwang-Jea Kim

**Affiliations:** DTR Co., 103, Sanmakgongdan buk 11-gil, Yangsan-si, Gyeongsangnam-do 626-120, Korea; jongyoung.lee@dtrkorea.co.kr

**Keywords:** polyamide66(PA66), glass fiber, monoethylene glycol(MEG), hydrolysis, mechanical property, pyrolysis-gas chromatography/mass spectrometry (py-GC/MS)

## Abstract

Polyamide66 (PA66) hydrolysis affects the mechanical properties of Polyamide66/glass fiber (PA66/GF) composites. We investigated the effects of monoethylene glycol (MEG) on the degree of hydrolysis and mechanical properties of four different commercial PA66/glass fiber composites. Using pyrolysis-gas chromatography/mass spectrometry (py-GC/MS), we identified the byproducts of PA66 composite hydrolysis: carboxylic acid and alkylamine substances. The degree of hydrolysis increased as the immersion time in MEG increased. However, the tensile and flexural properties decreased due to hydrolysis. The tensile strength decreased by 42–45%; however, elongation increased by 23–63%. When PA66 absorbs MEG at 130 °C, the materials molecular chains’ bonding force decreased, resulting in increased elongation.

## 1. Introduction

In developed countries, automobiles’ fuel efficiency and CO_2_ emissions have witnessed stronger regulatory terms. To achieve better fuel efficiency, the application of light-weight materials is on the rise in the automotive industry based on the Corporate Average Fuel Economy (CAFE) regulations [1,2]. Countries around the world are proposing solutions for lowering CO_2_ emission through improved energy efficiency levels in automobiles. Among which, lighter weight vehicles are attracting interest as one of the key solutions in the industry. In this context, plastics are being used more than ever, based on their characteristics; eco-friendly and better gas mileage. More stringent environmental regulations have driven active development of new recycling technologies, making plastic materials more attractive.

Polyamides (PA) represent one of the most recyclable engineering plastic materials used for light-weight vehicles. PA—an aliphatic polyamide—is a polymer of amide (-CONH-) linked monomers, which was first developed in 1935 [3]. PA66′s characteristics have made it a subject of ongoing studies [4,5,6,7,8,9,10,11,12,13,14]. Its main feature is the long-term durability against high temperatures (100–150 °C), thanks to its high level of crystallinity, heat resistance, wear resistance, and mechanical strength.

Other plastic materials with different structures such as Polyacetal (POM), Polycarbonate (PC), Polyethylene terephthalate (PET), and Polyphenylene oxide (PPO) are also widely used [15,16,17].

To identify the correct properties, additives like compatibilizers, lubricants, anti-oxidants, stabilizers, and flame retardants are used [18].

Generally, glass fibers (GF) have been used as fiber-type reinforcing fillers for plastic materials. However, recent trends indicate that a wider variety of materials are being applied for this purpose, including aramid fiber (AF), carbon fiber (CF), carbon nano tube (CNT), etc. [19,20]. GF improves strength and heat resistance, therefore, plastic materials reinforced with GF are highly resistant to external impact, and their tensile strength is largely improved [19].

Materials required for engine mount nozzles should have high tensile strength, and those exposed to glycol and moisture should be hydrolysis resistant. Certain characteristics must be met for component materials exposed to under-hood heat (150 °C), extreme environmental conditions (temperature = −40 °C), impact, corrosion from potassium chloride, fluids, or windshield wiper fluid, etc.

Enhancing hydrolysis resistance of PA could minimize mechanical strength drop in materials, allowing its incorporation as a component for high performance vehicles. PA66, which satisfies these requirements, should be developed. In fact, industrial studies aimed at developing such materials with hydrolysis resistance are ongoing [15,16,21,22,23,24,25,26,27]. Currently, several grades of hydrolysis resistant PA66/GF composites exist on the market; however, no direct scientific comparison of their hydrolysis resistance properties has been conducted, and no verification or correlation has been established between their degree of hydrolysis resistance and mechanical property changes. We have previously determined PA66/GF composite’s degree of hydrolysis using a pyrolysis-gas chromatography/mass spectrometry (py-GC/MS) analysis [27].

In this study, we determined the degree of hydrolysis resistance of four different PA66/GF composites immersed in monoethylene glycol (MEG), and compared its effects on mechanical property changes.

## 2. Experiment

### 2.1. Material

The materials used in this test are DTRamid composite materials (PA66/GF30, and WG30DTR HSLR; hereinafter referred as D) from DTR. A comparison was made with materials of the equivalent commercial grade including Zytel 70G30 HSLR (DuPont; hereinafter referred as Z), KDG 1030 (KOPLA; hereinafter referred as K), and A3WG6 HRX (BASF; hereinafter referred as A). These materials are hydrolysis resistance grade GF 30% reinforced PA66 composites. The composition of each material supplied is summarized in Table 1.

### 2.2. Sample Preparation

Composite materials were dried at 90 °C for 4 h, and maintained moisture levels lower than 0.05%, before they were loaded onto an injection molding machine. Fifteen specimens (ISO dumbbell-shaped type) were prepared using an injection molding machine (Hydraulic type, Ø30, Woojin Plaimm Co., Ltd., Chungbuk-do, Korea) at 85 °C mold temperature, 10 bar back pressure, 70 bar maximum injection pressure, 55 bar holding pressure, and 25 s of cooling time.

### 2.3. Immersion in MEG Solution

The test specimens were immersed into a flask-shaped reactor filled with MEG solution (Dex-cool, monoethylene glycol, ACDelco), at 130 °C for 504 h, and 1008 h, consecutively, and mechanical property changes were measured.

### 2.4. Mechanical Properties

Tensile strength: tensile strength, tensile modulus, and percentage elongation were measured for the specimens cross-head speed at 5 mm/min, using a universal testing machine (Instron 5967) in accordance with ISO 527-1 and 2 method.

Impact strength: as specified in ISO 179-1, the specimens’ impact strength was measured using an impact tester (CEAST 9050). The measurement was carried out with unnotched specimens using the Charpy method. Formula (1) was applied for the calculations.

(1) $$\mathrm{IS}=\frac{E_{C}}{T\times W}\times 10^{3}\;\left[\mathrm{KJ}/m^{2}\right]$$

where: IS: Charpy impact strength (KJ/m^2^); E_C_: corrected energy (KJ); T: thickness (m); and W: width (m).

For tensile and impact strengths, the average values from 5 specimens were used for each condition.

### 2.5. Hydrolysis (Molecular Structure Change) Observation

A pyrolysis-gas chromatography/mass spectrometry (py-GC/MS, Frontier Lab PY-3030D) was used to analyze the degree of hydrolysis and changes in the molecular structure inside PA66 composites under each condition. The pyrolysis was performed at 700 °C for 5 s, and the oven temperature was held at 40 °C for 5 s, and then increased incrementally to 320 °C at 10 °C/min.

## 3. Results and Discussion

### 3.1. Changes in Mechanical Properties After Immersion in MEG

After 1008 h of immersion in MEG, the tensile strength of D, Z, K, and A decreased from 178 MPa, 185 MPa, 171 MPa, and 167 MPa to 100 MPa, 102 MPa, 94 MPa, and 96MPa, respectively. These data indicated a drop in the tensile strength of the PA66/GF composites exposed to MEG at 130 °C. The rates of decrease in the tensile strength of D, Z, K, and A were shown to be −43.82%, −44.86%, −45.03%, and −42.51%, respectively. Post-immersion comparison showed that the tensile strengths of D and Z were higher than those of K and A.

Under the same conditions, the tensile modulus of D, Z, K, and A decreased from 9551 MPa, 9494 MPa, 9078 MPa, and 9039 MPa to 4646 MPa, 5022 MPa, 5099 MPa, and 4561 MPa, respectively. The decrease rates of the tensile modulus for D, Z, K, and A were −53%, −49%, −55%, and −50%, respectively. On the other hand, the percentage elongation of D, Z, K, and A increased from 3.1%, 3.5%, 2.9%, and 3.3% to 4.3%, 4.4%, 3.5%, and 5.4%, respectively.

Lissi and Chaupart similarly reported that PA66 composites show decreased mechanical properties after hydrolysis [28,29].

**Figure 1:**
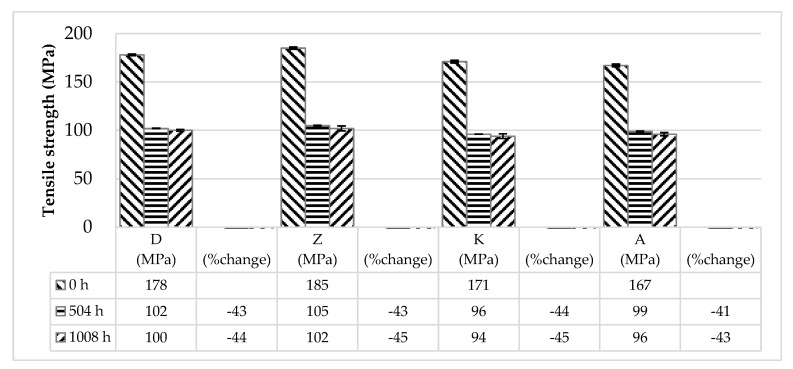
Tensile strength changes of various glass fiber-filled polyamide66 composites with respect to monoethylene glycol (MEG) immersion time increase at 130 °C.

The mechanical properties of various glass fiber filled polyamide66 composites, before and after immersion in MEG (1008 h) are as shown in Table 2. The tensile strength changes of various glass fiber-filled polyamide66 composites, with respect to the MEG immersion time increase, at 130 °C are shown in Figure 1.

**Figure 2:**
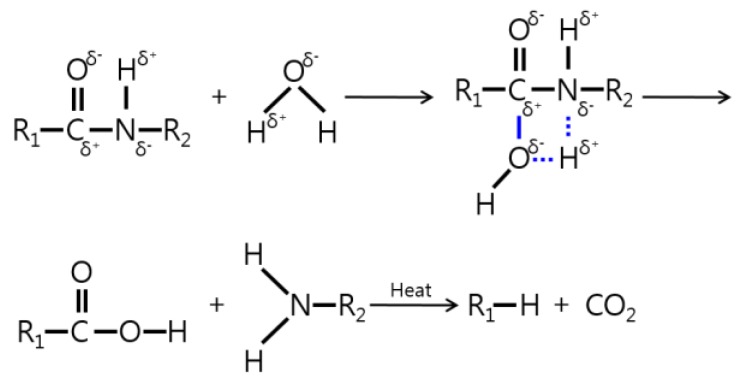
Hydrolysis of polyamide (adopted from Reference [37]).

Polymer absorption properties proportionally increase with free volume in the composite [30,31], and hydrogen bonds with polar atoms in PA66 [32,33]. PA66 absorbs moisture as the oxygen (O) and nitrogen (N) atoms in the chain form hydrogen bonds with the hydroxyl group. Hydrolysis degrades the properties of PA66/GF composites by breaking the PA66 chains [34,35,36]. Figure 2 illustrates the PA hydrolysis reaction mechanism [37]. Under moisture conditions, the –OH group is absorbed onto the polar functional group (C=O, -NH) of PA66 molecules, through a dipole–dipole interaction leading to hydrolysis [1,9,23,24,25,26,27,28,29,30,31,32,33,34,35,36,37,38,39,40,41,42,43,44,45]. This caused a decrease in tensile strength, which was consistent with our test results. The molecular structure of PA66 changes due to recrystallization of the semi-crystalline PA66 [46,47,48], during which the glass transition temperature (Tg) is 72 °C [18]. The recrystallization effect is more significant when PA66, exposed to MEG, dries at 100, 150, and 200 °C. As the drying temperature increases, and exposure time increases, the tensile strength also increases [24].

Hydroxyl groups behave as plasticizers among the PA chains and on the GF surface, thereby weakening the dipole–dipole interaction between chains and with GF [10,38,39,40,41,42,43]. Moisture absorption increases PA chains’ volume and mobility, thus weakening the entanglement and bonding between the molecules [37,44,45]. Once moisture is absorbed in GF-filled composites, GF reinforcement is reduced, i.e., lower tensile strength and higher percentage elongation. This also reduces the stress transfer capability between the matrix resin and GF interface [49]. These data are in accordance with our test results, and support the percentage elongation increase that was witnessed.

### 3.2. Py-GC/MS Observation

**Figure 3:**
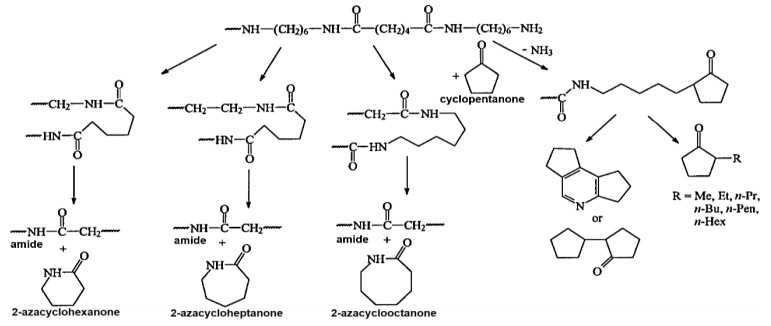
PA66 degradation mechanism and its byproducts (adopted from Reference [56]).

Py-GC/MS is widely used for qualitative and quantitative analyses of polymer composites and for obtaining information on molecular structures [50]. Hydroxyl groups are known to react and hydrolyze PA66 polar functional group (C=O, -NH) [21,22]. The pyrolysis byproducts of PA66 are cyclopentanone, hexamethylenediamine, and cyclic monomer [51,52,53,54,55]. The PA66 pyrolysis mechanisms, demonstrated by MacKerron and Gordon [56], are shown in Figure 3. PA66 chains undergo chain scission and reorganization to form cyclic monomers [56,57]. We showed that cyclopentanone, hexamethylenediamine, etc. are byproducts of PA66 pyrolysis [27].

Chromatograms of the PA66 pyrolysis byproducts before and after the immersion were compared using py-GC/MS. The results showed that as immersion time increased, PA66 byproducts increased as well, these included cyclopentanone (retention time (r.t.) 5m, ①), 2-N-Hexylaziridine (r.t. 8.6m, ②), 1-Decanol (r.t. 9.5m, ③), 1,6-Hexanediamine (r.t. 11.6m, ④), 3-None-1-ol(r.t. 13.4m, ⑤), Adiponitrile (r.t. 13.5m, ⑤), 1-Methyl-3-formlindole (r.t. 17.8m, ⑥), 2-Azacyclotridecanone (r.t. 21.5~31.3m, ⑦), and Hexadecanenitrile (r.t. 30.7m, ⑧). The decomposition products of PA66 composites are shown in Table 3.

**Figure 4:**
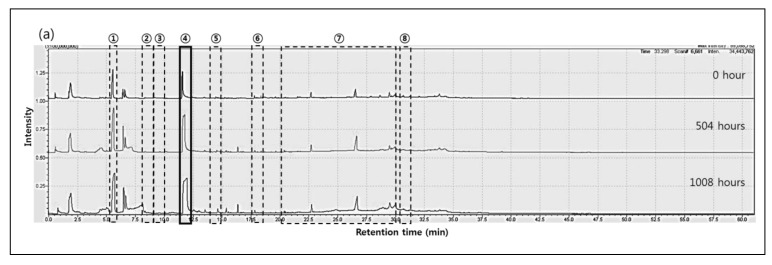

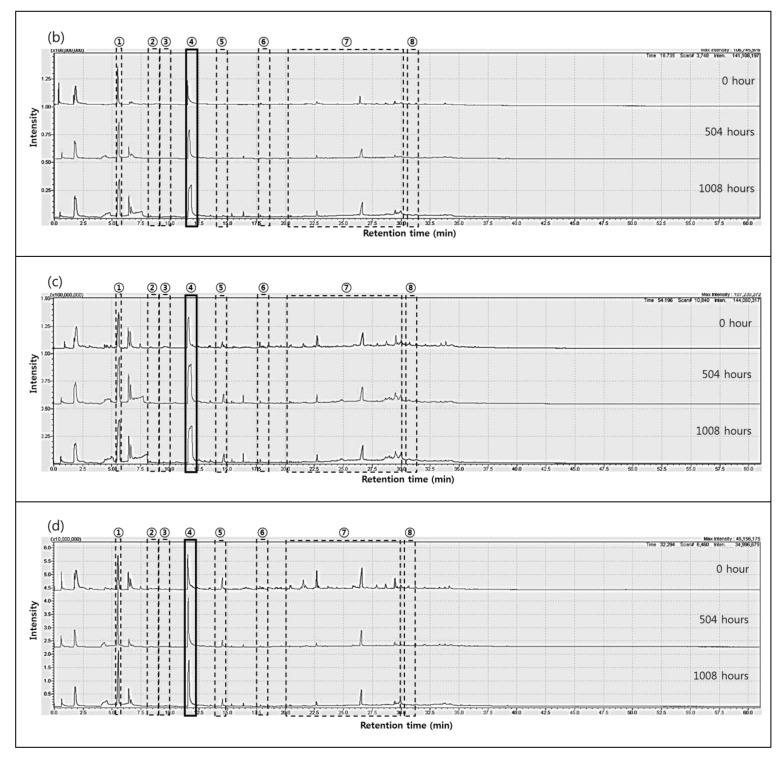
Pyrolysis-gas chromatography/mass spectrometry (py-GCMS) pyrograms of composites (**a**) D, (**b**) Z, (**c**) K, and (**d**) A with respect to immersion time.

The above result showed that scission of the PA66 chains to low molecules resulted from the hydrolysis reaction between MEG and PA66 chains. In other words, as the hydroxyl groups of MEG reacted with the polar functional groups (C=O, -NH) of PA66, carboxylic acid and alkylamine substances increased. Figure 4 shows py-GC/MS pyrograms of composites (a) D, (b) Z, (c) K, and (d) A with respect to the immersion time.

**Figure 5:**
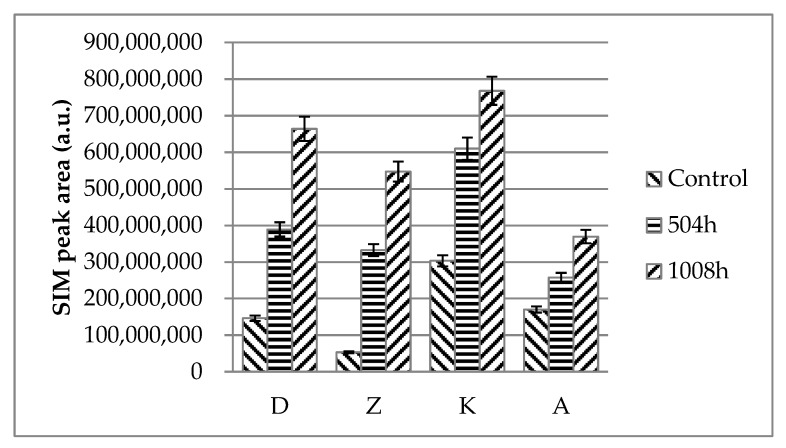
Pyrolysis-gas chromatography/mass spectrometry-selected ion monitoring (py-GC/MS-SIM) peak area corresponding to 1,6-hexanediamine in PA66/GF composites with respect to immersion time.

The 1,6-Hexanediamine ions (*m*/*z* = 30) were selected for quantitative analysis as they had the highest strength in the captured mass spectrum, and showed the highest selectivity. The analysis results of the 1,6-Hexanediamine ions using the GC/MS-selected ion monitoring (SIM) mode are shown in Figure 5. As immersion time increased (0 to 1,008 h), more 1,6-Hexanediamine ions were detected from D, Z, K, and A. This provides proof that hydroxyl groups of moisture (MEG) react with PA66 chains via hydrolysis, which in turn decomposed to 1,6-Hexanediamine.

Overall, the retention time and chemical structures of PA66 composite materials pyrolysis byproducts are listed in Table 3. MacKerron and Gordon [56] reported byproducts of the PA66 pyrolysis products, which were consistent with our observations as shown in Figure 5.

Regarding D and Z, we observed increase of hydrolysis byproducts from all regions (①, ②, ③, ④, ⑤, ⑥, ⑦, and ⑧) as shown in chromatographs; however, K and A did not show significant changes except for region ④ (1,6-Hexanediamine). It is not clear why they did not show significant changes at this stage.

The correlation of SIM peak area analysis with hydrolysis resistance and tensile strength will be discussed in the next issue following the accumulation of more data.

## 4. Conclusions

This study has made a comparative analysis of hydrolysis resistance for various PA66/GF composites (D, Z, K, and A) by increasing immersion time in MEG at 130 °C.

Py-GC/MS analysis results showed that PA66/GF composites reacted with MEG hydroxyl groups and decomposed into low molecular structures via hydrolysis. The results also showed that increased immersion time (upto 1008 h), led to more decomposed low structure molecules, and their tensile strength proportionally decreased.

Hydrolysis of PA66 affected the mechanical property changes of PA66/GF composites. After immersion, the tensile strength of D, Z, K, and A dropped to 100MPa, 102MPa, 94MPa, and 96MPa, respectively. D and Z maintained higher tensile strength compared to K and A. The tensile strength drop rates were measured to be around −42–−45% (i.e., −43.82% (D), −44.86% (Z), −45.03% (K), and −42.51% (A)). Percentage elongation increased to ~30–60% (i.e., 40% (D), 28% (Z), 23% (K), and 63% (A)).

Overall, the hydroxyl groups in MEG activated hydrolysis of the PA66 chain reaction, decreased its tensile strength, weakened the entanglement between PA66 chains and between PA66 and GF surfaces, thereby increasing the percentage elongation.

## Figures and Tables

**Table 1:** The composition of glass fiber 30% filled PA66.

	D	Z	K	A
PA66 (wt%)	66.78 (± 0.61)	68.81 (± 0.41)	66.24 (± 0.88)	66.92 (± 0.54)
GF (wt%)	31.52 (± 0.76)	29.25 (± 0.38)	30.35 (± 0.18)	30.78 (± 0.39)
Additive (wt%)	1.71 (± 0.15)	1.95 (± 0.03)	3.41 (± 0.7)	2.30 (± 0.15)

**Table 2:** Mechanical properties of various glass fiber-filled polyamide66 composites, before and after immersion in monoethylene glycol (MEG) (1008 h) at 130 °C.

		Tensile Strength (MPa) [5 mm/min]	Tensile Modulus (MPa) [5 mm/min]	Tensile Elongation (%) [5 mm/min]	Impact Strength (kJ/m^2^) [unnotched]
D	Before	178	9551	3.1	66.04
	After	100	5099	4.3	69.42
	% change rate	−44	−47	+40	+5
Z	Before	185	9494	3.5	76.62
	After	102	4646	4.4	73.97
	% change rate	−45	−51	+28	−3
K	Before	171	9078	2.9	61.09
	After	94	5022	3.5	67.76
	% change rate	−45	−45	+23	+11
A	Before	167	9039	3.3	77.2
	After	96	4561	5.4	88.43
	% change rate	−43	−50	+63	+15

**Table 3:** The decomposition products of PA66 composites.

Region	Retention Time (min)	Product	Chemical Structure
①	5.5	Cyclopentanone	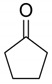
②	8.6	2-N-Hexylaziridine	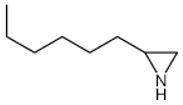
③	9.5	1-Decanol	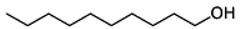
④	11.6	1,6-Hexanediamine	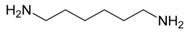
⑤	13.4	3-None-1-ol	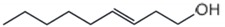
	13.5	Adiponitrile	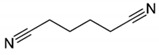
⑥	17.8	1-Methyl-3-formlindole	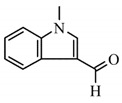
⑦	21.5 22.7 25.9 26.6 28.7 29.5 30.0 31.3	2-Azacyclotridecanone	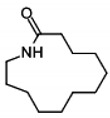
⑧	30.7	Hexadecanenitrile